# Effectiveness of exercise on fall prevention in community-dwelling older adults: a 2-year randomized controlled study of 914 women

**DOI:** 10.1093/ageing/afad059

**Published:** 2023-04-23

**Authors:** Toni Rikkonen, Reijo Sund, Heli Koivumaa-Honkanen, Joonas Sirola, Risto Honkanen, Heikki Kröger

**Affiliations:** Kuopio Musculoskeletal Research Unit (KMRU), University of Eastern Finland Kuopio, Finland; Kuopio Musculoskeletal Research Unit (KMRU), University of Eastern Finland Kuopio, Finland; Institute of Clinical Medicine (Psychiatry), University of Eastern Finland, Kuopio, Finland; Mental Health and Wellbeing Center, Kuopio University Hospital, Kuopio, Finland; Kuopio Musculoskeletal Research Unit (KMRU), University of Eastern Finland Kuopio, Finland; Department of Orthopaedics, Traumatology and Hand Surgery, Kuopio University Hospital, Kuopio, Finland; Kuopio Musculoskeletal Research Unit (KMRU), University of Eastern Finland Kuopio, Finland; Kuopio Musculoskeletal Research Unit (KMRU), University of Eastern Finland Kuopio, Finland; Department of Orthopaedics, Traumatology and Hand Surgery, Kuopio University Hospital, Kuopio, Finland

**Keywords:** exercise, fall prevention, fall injury, fracture, aging, older people

## Abstract

**Background:**

Communal exercise interventions may help prevent falls and injuries. However, pragmatic trials demonstrating the effectiveness of such strategies are sparse.

**Methods:**

We determined whether a cost-free 12-month admission to the city’s recreational sports facilities including initial 6 months of supervised weekly gym and Tai Chi sessions decreases the number of falls and related injuries. The mean (SD) follow-up time was 22·6 (4.8) months in 2016–19. A total of 914 women from a population-based sample with a mean age of 76.5 (SD 3.3, range 71.1–84.8) years were randomized into exercise intervention (*n* = 457) and control (*n* = 457) groups. Fall information was collected through biweekly short message (SMS) queries and fall diaries. Altogether 1,380 falls were recorded for the intention-to-treat analysis, with 1,281 (92.8%) being verified by telephone.

**Results:**

A 14.3% fall rate reduction was detected in the exercise group (Incidence rate ratio (IRR) = 0.86; CI 95% 0.77–0.95) compared with the control group. Approximately half of the falls caused moderate (*n* = 678, 52.8%) or severe (*n* = 61, 4.8%) injury. In total, 13.2% (*n* = 166) of falls (including 73 fractures) required medical consultation with a 38% lower fracture rate in the exercise group (IRR = 0.62; CI 95% 0.39–0.99). Overall, the greatest reduction of 41% (IRR = 0.59; CI 95% 0.36–0.99) was observed in falls with severe injury and pain.

**Conclusions:**

A community-based approach for a 6-month exercise period combined with a 12-month free use of sports premises can reduce falls, fractures and other fall-related injuries in aging women.

## Key Points

- A two-year randomized controlled fall prevention study on community-dwelling older women.
- The greatest reduction was observed in falls with severe injury and pain.
- Community-driven fall prevention with light- or moderate-intensity exercise group programmes can reduce falls and fall injuries on a municipal scale.

## Introduction

The global proportion of people over 60 years is expected to double between 2000 and 2050 [1]. Depending on population health dynamics, this may cause a significant societal burden [2, 3]. Falls can cause substantial cost [4, 5], while reduction of falls and related injuries have been shown in fall prevention and exercise interventions [6–9] as well as in group- and home-based exercise programmes, including home safety interventions [10, 11]. Rates for women in non-fatal fall-related injuries are generally higher, for fractures about twice higher than in men [12] making gender-specific fall prevention studies a reasonable option. However, the number of fall-induced deaths among older Finnish adults has been increasing considerably [13].

Community-wide effects of fall prevention in the aging population warrant large-scale studies. Thus, pragmatic randomized controlled trial (RCT) studies with societal fall prevention strategies showing real-world effectiveness should be conducted. Population-based studies involving city populations could indicate whether physical exercise can provide a meaningful and tangible tool for fall prevention in urban environments, where the majority of municipal services are within reach.

However, the use of such services may be more appealing if they involve group activities. As participation in physical activities with a group of similar age and gender has promoted better compliance, it should be considered as a part of communal strategies in fall prevention [14].

In this study, we present the effectiveness of fall prevention for primary and secondary outcomes of falls, fall incidences, fall types, fall-related fractures and clinical measurements in community-dwelling aging women. We hypothesized that a 6-month introduction to physical activities combined with free use of all municipal sports premises could prevent falls and related injuries.

## Methods

### Study design

Kuopio Fall Prevention Study (KFPS) is a randomized controlled study conducted in cooperation with the municipality of Kuopio, with a population of ~120,000 inhabitants in eastern Finland. All the participants living in Kuopio urban area, born between 1932 and 1945, were invited through mass mailings using their home addresses. Institutional long-term care addresses were excluded. The trial is registered with ClinicalTrials.gov (NCT02665169). For more details on study methods, see the study protocol [15] and Appendix 1.

### Participants, randomization and masking

Between 1 January 2016 and 31 March 2016, an information letter was sent to 4,262 home dwelling women living within a 10-km radius of the city centre, asking their willingness to participate in the 2-year randomized exercise trial, resulting in 1,600 responses. The exclusion criteria were self-reported unstable angina pectoris, severe pulmonary disease, at least moderate dementia, or being non-ambulatory. After a phone review, a total of 1,208 invitations were sent. Of these, a total of 914 women participated in the baseline clinical measurement. From 14 March 2016 to 7 April 2017, randomization to exercise intervention (*n* = 457) or control groups (*n* = 457) was carried out. After the 1:1 block randomization of all participants (*N* = 914), randomization codes were concealed until the completion of baseline visit. After baseline, the study was conducted as open label trial.

### Primary and secondary outcomes

We studied the effect of an exercise intervention on the primary outcome, i.e. falls. In addition, falling mechanisms, injurious falls and fractures were analysed as the secondary outcome. All outcomes shared the same inclusion criteria (of tripping, slipping, syncope or similar event from less than 1-m height). The mean (SD) follow-up time for falls was 22.6 (4.8) months, with 1716.4 person-years. Recording of fall was done using cellular phone-based automated biweekly short message service (SMS) and subsequent phone interview. Thus, falls were verified within 2 weeks of the incident by a structured phone interview to provide details of fall mechanism, location, terrain, level of injury and pain (none/moderate/severe), medical treatment and induced fear of fall. In addition, the self-administered study diary was used as a backup method for the fall recording in 3-month intervals, including falls and time spent in physical activities. Fall risk estimates covered the time from the baseline up to the last contact date when any confirmed fall information, i.e. the last SMS response, returned study diary or study visit. ‘Outdoor falls’ required location with seasonal exposure to the elements. ‘Indoor falls’ definition required standard climate conditions. Self-reported fall types that are subject to individual interpretation, miscellaneous or unclear cause were categorized as unspecified. Fracture analysis was based on hospital discharge registry and patient perusals with a mean (SD) follow-time of 23.4 (3.8) months.

### Clinical measurements

**Figure 1:**
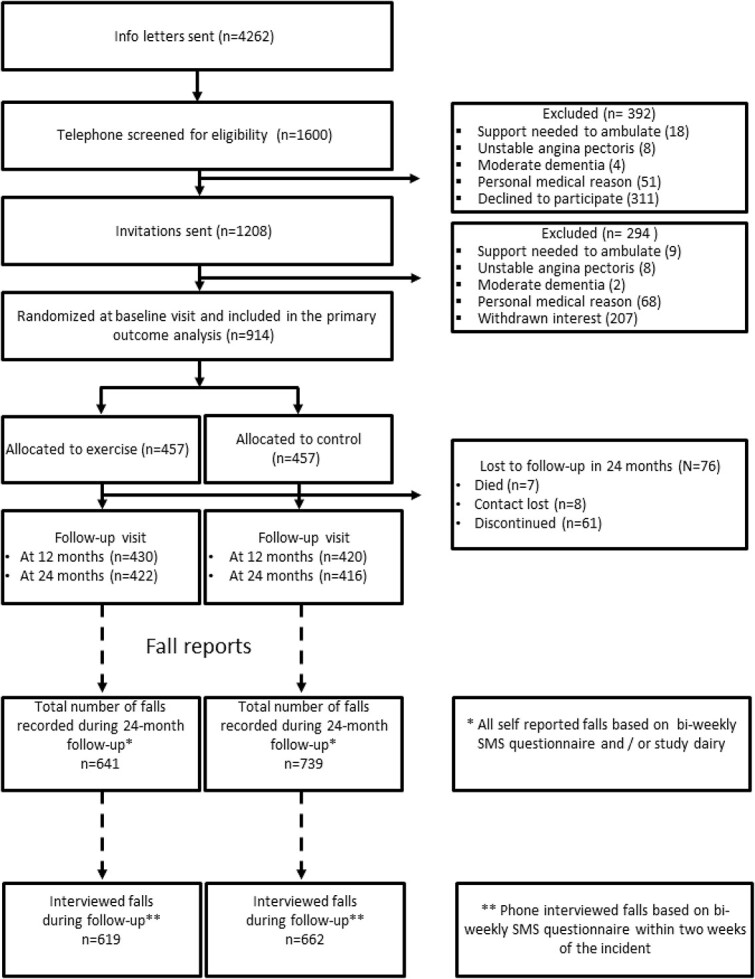
The study recruitment, screening and participation.

Clinical measurements were accomplished in Kuopio Musculoskeletal Research Unit at baseline for 914 (100%), and at 12- and 24-month follow-ups for 850 (93.0%) and 838 (91.7%) women, respectively (Figure 1). At each visit, weight, height, waist circumference and bone mineral density (BMD) from proximal femur and total body were measured. Functional measurements included single-leg stance, squat test, postural sway, isometric leg extension strength and maximal grip strength. See Appendix 1 for details.

### Exercise intervention and use of city premises

The women randomized to exercise intervention were allocated to 27 groups, each including 15–18 attendees. Same groups were maintained in both Tai Chi (‘Taiji’) and gym sessions. No additional support (such as motivational reminders, etc.) was given to women during the trial. The control group received education on fall prevention at the baseline visit and was free to pursue their personal activities as before. The intervention groups were provided with a personal electronic access card for free access to all the city exercise premises including swimming halls, gyms and other sports premises administered by the municipality for the first 12 months. At baseline visit, participants were verbally informed about the possibility to use any of the municipal exercise premises for free, in addition to the scheduled sessions, without any obligation to do so. The card was also obtainable from municipal offices by anyone with a pensioner status for an annual fee of 65 euros.

In addition to free use of premises, supervised exercise intervention was carried out during the first 6 months, aiming to improve muscle strength focusing on lower limbs, postural balance, active range of motion and joint mobility. The protocol included a 1-hour circuit type gym session and a 1-hour Tai Chi session each week, with a warm-up and 50 minutes of training. The adherence was measured by women’s participation to supervised sessions, based on logging data of the access cards. Group exercises were discontinued after the initial 6 months. However, women who wanted to continue gym training or Tai Chi at their own expense were not restricted from doing so. No individual modifications were done to training protocol during the intervention. However, in case of health restrictions, women were free to adapt the training with the instructors in a way they felt reasonable and pain-free.

### Gym

All gym sessions were supervised by two physiotherapists for the initial 6 months. The aim of the first four visits (weeks) to the gym was to familiarize study subjects with the safe and appropriate use of the weight training equipment. No free weights were used. The training prioritized postural muscle groups of legs (quadriceps/hamstring), back (lower/upper) and trunk, with additional workouts for the chest and arms. Starting weights were based on the participant’s results of a 4-week tutorial and prediction of one repetition maximum (1RM) (Borg’s scale) [16]. The number of repetition (12–15 per set) was decreased (to 8–12) after 6 weeks. The estimated training intensities (%1RM) for the initial 6-week strength endurance period and the following 12-week hypertrophy period were 65–70% and 70–80%, respectively. The warm-up session included light stretching and mobility exercises. The exercise protocol has been described in detail previously [17].

### Tai chi

The Tai Chi course—designed and executed in cooperation with a professional Tai Chi instructor—aimed to reduce falls by improving balance, posture and active range of motion through the variations of classic Tai Chi exercises. The protocol progressed from simple postural control, body weight shifting and static balance training to the coordinated series of Tai Chi movements which eventually allowed independent training. Particular attention was given to lower limb strength, postural muscles and coordination. A standard Tai Chi protocol was issued for all participants throughout the study.

### Sample size and statistical analysis

Previous meta-analyses and RCTs reported heterogeneous dropout rates depending on factors such as geographic location, sociodemographic status, and duration and intensity of the protocol [8, 18, 19]. Our dropout estimate was set to 20% for the exercise and 16% for the control group. We estimated, with a 5% error margin, that 341 women in both groups would be required to provide an 80% power of detecting 31% fall reduction with a 30% general fall incidence during the follow-up. According to the estimated 3% annual mortality and 20–16% dropout percentages for exercise and control samples, respectively, the group sizes were calculated as (341/0.77) + (341/0.81) = 442 + 420. Adjusted for 80% compliance with mixed effects, the total group size was estimated to be 1,078. Due to a slight under-recruitment, the study was 164 (15%) women short on the prespecified sample size calculation with a total of 914 women.

The Fall risk analyses were conducted according to the intention-to-treat by including all women up to their last confirmed fall information, regardless of their exercise adherence. The predefined statistical analysis was conducted by an unblinded researcher who had access to the outcome data. All available cases were included in the follow-up analyses of clinical measurement data. Chi-square test was used to compare proportion of women with any fall and fall injury between groups. Incidence rate ratios (IRR) were obtained using Poisson regression analysis with a 95% confidence interval (CI) for multiple falls and contributing fall mechanisms. Fracture IRR and survival curves were analysed using the Poisson regression model and Kaplan–Meier curves with Log-rank test, respectively.

## Results

### Participants and adherence

The mean age of the women at baseline was 76.5 (3.3). The total number of dropouts was 76 (8.3%) including deaths (*n* = 7), withdrawn consents (*n* = 61) and women lost to follow-up (*n* = 8). The mean participation rate for the supervised sessions had 70.4–79.6% coverage of the total sessions held depending on the group. The proportion of women attending over 80% of the supervised sessions was 61.9% (*n* = 283). On the contrary, 14 out of 457 women in the exercise group were non-compliant with 0 visits. The total number of supervised sessions had some group-specific variation between 23 and 26 weeks, due to practical reasons such as national holidays. Baseline characteristics and clinical measurements with their relative changes during the follow-up are presented in Table 1. No serious adverse events were reported during the follow-up that could be directly related to training sessions. For adverse events description, see Appendix 1.

**Table 1:** Baseline characteristics of the study population with the follow-up change of bone densitometry and functional tests

Baseline characteristics	Control *n* = 457 (SD)	Intervention *n* = 457 (SD)
Follow-up time, years	1.87 (0.41)	1.89 (0.40)
Age, years	76.6 (3.2)	76.4 (3.3)
Height, cm	159.4 (5.5)	158.4 (7.4)
Weight, kg	68.8 (11.8)	69.5 (12.8)
BMI, kg/m^2^	27.0 (4.4)	27.5 (4.6)
Current smoker, %	13 (2.8)	4 (0.9)
**Bone densitometry and functional tests**		
Femoral neck BMD, g/cm^2^	*0.845 (.128)*	*0.840 (.127)*
- Change over time^a^ (%)	*−1.52 (3.6)*	*−1.52 (3.4)*
Femoral neck T-Score (NHANES III)	*−1.4 (0.9)*	*−1.4 (0.9)*
Prevalence of		
- Osteopenia (%)	*219 (47.9)*	*253 (55.3)*
- Osteoporosis (%)	*40 (8.9)*	*29 (6.3)*
Total body lean mass, kg	*38.9 (4.2)*	*39.0 (4.3)*
- Change over time^a^ (%)	*−0.7 (2.9)*	*−0.5 (2.9)*
Total body fat mass, kg	*27.5 (8.5)*	*28.0 (8.8)*
- Change over time^a^ (%)	*−0.06 (8.3)*	*−0.6 (10.4)*
Grip strength, kg	*27.2 (4.9)*	*26.9 (5.4)*
- Change over time^a^ (%)	*−1.8 (16.2)*	*−1.1 (11.4)*
Leg extension strength, *N*	*303.8 (69.4)*	*307.0 (71.9)*
- Change over time^a^ (%)	*−3.8 (15.1)*	*−1.7 (14.8)*
Timed Up and Go, seconds	*9.7 (1.9)*	*9.8 (2.0)*
- Change over time^a^ (%)	*13.7 (20.1)*	*14.5 (23.5)*
One leg stance, seconds	*15.5 (11.2)*	*15.2 (11.4)*
- Change over time (%)	*−0.3 (8.1)*	*0.8 (8.3)*
Able to squat down and get up without support (%)		
- At Baseline	*97.3*	*99.1*
- 24 months^a^	*97.6*	*98.8*
Postural sway		
Normal stance eyes open, mm^2^	*188.3*	*173.9*
- Change over time^a^ (%)	*16*	*13.5*
Normal stance eyes closed, mm^2^	*297.5*	*265.3*
- Change over time^a^ (%)	*20.4*	*24.8*
Semi-tandem, eyes open, mm^2^	*270.5*	*254.2*
- Change over time^a^ (%)	*11.2*	*14.5*
Semi-tandem, eyes closed, mm^2^	*602*	*546.5*
- Change over time^a^ (%)	*17.8*	*25.8*

^a^Proportional change over 24 months by available follow-up DXA measurement (*n* = 831).

### Falls and injuries

A total of 1,380 falls were registered during the 24 months. Of these, 1,281 (92.8%) were reported through SMS, the rest being recorded from study diaries. The mean follow-up (SD) for the falls was 23.8 months. In total, 15 women did not respond to any fall inquiry and were excluded from the primary analyses. The proportion of phone interviewed falls was somewhat lower in the control group (89.6%) vs. the intervention group (96.6%). Total follow-up time was 1,716.4 person-years, during which 546 women had at least one fall (range 0–28) with a crude incidence of 804.0 falls per 1,000 person-years and a prevalence of 59.7% (Table 2).

**Table 2:** Characteristics and distribution of all falls (*n* = 1,380) and fall-related factors according to phone interviews (*n* = 1,281) with crude incidence rate ratio (95%CI) using the Poisson regression model (reference: Control group)

	Control N = 457 (100%)	Intervention N = 457 (100%)	Crude IRR (95%CI)	p-value
Fallers during follow-up, n (%)	278 (60.8)	268 (58.6)		0.474^a^
**All recorded falls, n (%)** Rate per 1,000 person years, (95CI%)	**739 (100%)** 865.0 (864.3–865.6)	**641 (100%)** 743.6 (742.7–744.5)	0.86 (.77–.95)	**0.004**
**Phone interviewed falls, n (%)** Outdoor falls Indoor falls	**662 (100%)** 492 (74.3) 170 (25.7)	**619 (100%)** 489 (79.0) 130 (21.0)	0.92 (.83–1.03) 0.98 (.87–1.11) 0.76 (.60–.95)	0.160 0.983 **0.016**
Used anti-skid devices on shoes	17 (2.6)	20 (3.3)	1.16 (.61–2.20)	0.647
Used ‘walking poles’ or similar support	59 (8.9)	44 (7.1)	0.74 (.50–1.10)	0.126
Reached forward with arms	226 (34.1)	211 (35.7)	0.92 (.77–1.11)	0.403
				
**Contributing fall mechanism, n (%)** Slipping Tripping Syncope/dizziness Stumbling Bicycle Cross country skies Misstep or moving platform (Train, bus, etc) Unspecified or cannot say	**662 (100%)** 211 (31.9) 221 (33.4) 28 (4.2) 52 (7.9) 18 (2.7) 36 (5.4) 41 (6.2) 55 (8.3)	**619 (100%)** 246 (39.7) 204 (33.0) 10 (1.6) 26 (4.2) 10 (1.6) 29 (4.7) 42 (6.8) 52 (8.4)	1.15 (.96–1.39) 0.91 (.75–1.10) 0.35 (.17–.73) 0.49 (.31–.79) 0.55 (.25–1.12) 0.80 (.49–1.30) 1.01 (.66–1.56) 0.94 (.64–1.37)	0.130 0.346 **0.005** **0.003** 0.129 0.362 0.954 0.727
**Terrain, n (%)** Street/floor/even surface Stairway Slope/hill Doorway/doorstep Forest/outdoor trail Unspecified or cannot say	**662 (100%)** 320 (48.3) 55 (8.3) 76 (11.9) 15 (2.3) 159 (24.0) 37 (5.6)	**619 (100%)** 288 (46.5) 46 (7.4) 104 (16.8) 17 (2.7) 141 (22.8) 23 (3.7)	0.82 (.70–.96) 0.76 (.52–1.13) 1.25 (.93–1.68) 1.04 (.52–2.01) 0.81 (.65–1.02) 0.57 (.34–.96)	**0.016** 0.179 0.138 0.921 0.069 **0.033**

^a^Pearson Chi-Square analysis.

Overall, the proportion of fallers (at least one fall) was similar in control (60.8%) and intervention groups (58.6%) during the follow-up. However, a 14.2% fall rate reduction (*P* = 0.004) was observed in the intervention group compared with the control group (Table 2) in multiple falls. Similar reduction was also seen in indoor (−24.5%) (*P* = 0.02) but not in outdoor falls (−3%), as well as in severely injurious (−40.7%, *P* = 0.043) and non-injurious falls (−15.7%, *P* = 0.046). Overall, 52.9% of the interviewed falls were reported at least moderately injurious, with 13.3% requiring medical attention (Table 3). In a partial analysis for the first year, 43.7% of the women fell covering 764 falls, with a similar proportion of fallers in the control (43.6%) and intervention (43.8%) groups. Although an 8.7% fall rate reduction was seen with 399 versus 365 falls in the control and intervention group, respectively (IRR = 0 0.91; CI 95% 0.79–1.05), this did not reach statistical significance for the first year alone (*P* = 0.2).

**Table 3:** The characteristics of fall injuries (*n* = 1,281) verified by phone interviews and fall-related fractures (*n* = 73) according to hospital discharge registries, with a crude incidence rate ratio (95%CI) using the Poisson regression model (reference: Control group)

FALL INJURIES	Control N = 457 (100%)	Intervention N = 457 (100%)	Crude IRR (95%CI)	**p-value**
**Women with injurious fall, n (%)**	**195 (42.8)**	**207 (45.3)**		0.464^a^
**Level of a self-reported fall injury, n (%)** Non-injurious Moderate injury Severe injury	**662 (100%)** 282 (42.6) 343 (51.8) 37 (5.6)	**619 (100%)** 260 (42.0) 335 (54.1) 24 (3.9)	0.84 (.71–1.0) 0.89 (.77–1.0) 0.59 (.36–.99)	**0.046** 0.139 **0.043**
**Event induced fear of fall (yes)**	150 (22.7)	128 (20.7)	0.84 (.67–1.07)	0.158
**Event required medical attention, n (%)**	93 (14.0)	78 (12.6)	0.83 (.61–1.12)	0.223
**Women with fall related fracture, n (%)**	39 (8.5)	24 (5.3)		**0.049** ^**a**^
**Number and type of fracture, n** Hip Clinical vertebrae Wrist Other	**45** 6 11 13 15	**28** 5 2 13 8	0.62 (.39–.99)	**0.047**

^a^Pearson Chi-Square analysis.

Nearly half (47.5%) of the interviewed falls occurred on the street, floor or similar even surfaces, with 17.7% (*P* = 0.016) fall reduction in the intervention group (Table 2). Presyncope/dizziness (−67.4%, *P* < 0.005) and stumbling (−50.6%, *P* = 0.003) were the only specific causes associated with fall reduction, although being responsible only for 9.1% of the total observed. In total, 53.6% of the women reported at least occasional fear of fall at baseline. Although the proportion of women with fear of fall was lower at end of the follow-up, it remained similar with 47.2% and 49.4% for intervention and control groups, respectively (*P* = 0.518).

**Figure 2:**
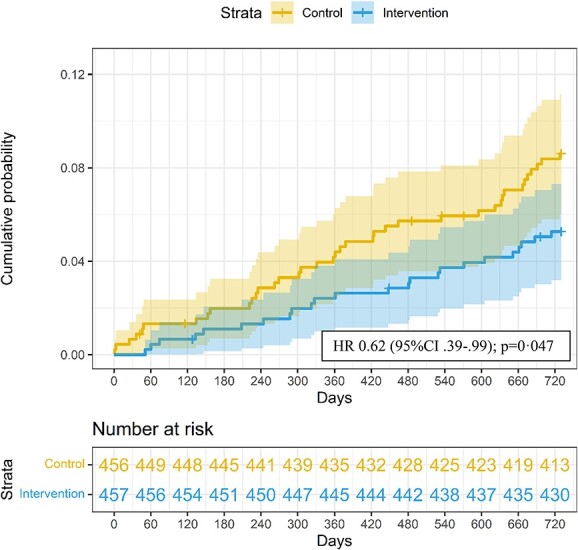
Cumulative probability of any fracture during the follow-up in the control (Yellow) and intervention (Blue) groups (Kaplan–Meier curves, log-rank).

Altogether 77 fractures caused by falls were observed in 66 women. The fractures caused by a vehicle (*n* = 3) and cancer (*n* = 1) were excluded. Out of these, 39 women belonged to the control and 24 to the intervention group (Table 3). Although the study was primarily designed for falls and fall injuries, the secondary analysis showed a 38% lower fracture rate in the intervention group with the Poisson regression model (IRR = 0.62; CI95% 0.39–0.99, *P* = 0.047) and the Kaplan–Meier model (Log-rank *P* = 0.047) (Figure 2).

### Clinical measurements

A complete 24-month follow-up data were available for 831 (90.9%) women. BMD or body composition results did not change during the follow-up. However, in functional tests, the leg extension strength decreased slightly less (−1.7 versus −3.8%, *P* = 0.05) and one leg stance time improved marginally (+0.8% versus −0.3%, *P* < 0.05) in the intervention group compared with the control group (Table 1). Women had similar reduction of 13.7 and 14.5% in TUG test speed between control and intervention group, respectively. In contrast, postural sway showed slight increase in all women. The prevalences of osteopenia and osteoporosis in the study cohort were 51.6 and 7.5%, respectively.

## Discussion

The KFPS is a large population-based fall prevention RCT targeted at older community-dwelling women with minimal exclusion criteria. Weekly Tai Chi and gym exercises were combined with free access to recreational facilities, aiming to reduce the number of falls in a 24-months follow-up. The proportion of women who had at least one fall or fall injury was similar in both groups. However, the fall and fall injury rates were lower in the intervention group. Thus, the intervention’s effect was seen on repeat falls, severe fall injuries and fractures. Overall, the intervention group had a reduction of 14.3% in their overall fall rate compared with the control group. Their indoor falls—comprising a quarter of the falls—were especially decreased (−25.6%). Altogether, falls with self-reported severe injury and pain (~41%) were reduced the most. Almost half of the falls occurred on even surfaces—either indoors or outdoors—and a majority of these (68.9%) were due to tripping and slipping, as expected. In general, women’s functional capacity was not affected during the follow-up, but leg extension strength decreased less, and one-leg stance time improved slightly in the intervention group, which may relate to the number of falls. In all, perhaps the approach of working through the community rather than just through healthcare alone to prevent falls may be a better strategy.

The main target was the reduction of falls by improving balance, muscle strength and functional capability by promoting physical activity. The findings suggest that the risk factors for indoor and outdoor falls may be different. Although higher fall reduction was observed indoors, the majority (69%) of the falls occurred outdoors. Nevertheless, indoor falls are a common cause of serious fractures such as of the hip, notably since frail older people stay more inside and are at higher risk for indoor falls [20, 21]. In this study, relatively few (13.2%) falls resulted medical care.

The strength of this study is the meticulous fall monitoring and interview within 2 weeks of the reported event. This probably ensured better recollection of the events and circumstances contributing to the falls—and resulted in capturing substantially more falls than anticipated. Previous studies have showed limitations to report falls that occurred during over the preceding 3–12 months [22, 23]. To our knowledge, this is the first communal exercise RCT targeted to the entire age cohort of older women, combined with an SMS fall reporting*.* However, there are no validation studies on this method that could estimate the additional benefit of the system. Further analyses are also needed to investigate the access card utilization outside the protocol and the cost-effectiveness in respect of fall injuries. In addition, corresponding studies with males should be conducted.

As the exclusion criteria were kept to a minimum, the training was designed with low to moderate intensity to suit most older women with average functional capability. Thus, we did not expect to see a change in terms of body composition or BMD. Nevertheless, slightly less reduced leg extension strength and improved one leg stance time—probably due to better muscle control in the exercise group—have been shown to be beneficial in fall prevention previously [24–26]. In order to have any significant osteogenic effect, the intervention would have to have been exceedingly more intensive [27–29], which in turn would have led to additional compliance and safety concerns. By keeping the intensity moderate, we were able to implement the intervention to this age cohort as broadly as possible. In addition, while the intervention utilized city premises, the exercise coach and Tai Chi instructor are not routinely available to all city residents. These factors place some limits on the generalizability of the study.

Several factors might potentially affect the study results. For practical reasons, we could only include blinding up to the baseline visit. Some of the study effects may also be influenced by RCT participation bias since only 21.4% of the invited women were recruited. Lastly, verifying SMS fall reports through phone interviews was appropriate to eliminate false positives but did not address false negatives. The number of phone interviewed falls was somewhat lower in the control group (89.6%) versus intervention group (96.6%). This is likely to cause some underreporting of falls in the control group, which may have led to a more conservative estimate in terms of fall prevention. However, this was not the case with fractures which were recorded from registries, regardless of self-reporting.

The fall prevention efficacy was as expected to differ from high-risk groups, such as hospitalized or institutionalized adults [30, 31]. Our recent comparison of health and social information between participants and non-participants of the KFPS trial showed that the women attending were better off for physical and mental well-being, functional capability and sociodemographic status than women on average in this region [32]. The prevalence of osteoporosis, but not osteopenia, was lower in this local cohort compared with some other populations [33, 34], although we have previously shown that the prevalence of osteoporosis is also low in this age group regionally [35]. Thus, healthier participants were likely to have more conservative fall and fall injury rate estimates than the general population of this age which may dilute the effect of this RCT for some degree. However, the results indicate that community-driven fall prevention, conducted in the urban environment, can be effective among women who are able and willing to participate in such programmes. The efficacy of similar health promotion in home-dwelling populations with poor functional capability or without interest in such activities remains unclear.

In conclusion, fall prevention with light- or moderate-intensity exercise group programmes can reduce falls and fall injuries among older women on a municipal scale. Broader implementation of such strategies and societal health promotion should be developed through municipal services and communities to achieve these aims in the future.

## Supplementary Material

aa-22-1748-File002_afad059

## Data Availability

The data underlying this article will be shared on reasonable request to the corresponding author.
