# Correction to: Effectiveness of exercise on fall prevention in community-dwelling older adults: a 2-year randomized controlled study of 914 women

**DOI:** 10.1093/ageing/afad084

**Published:** 2023-05-19

This is a correction to: Toni Rikkonen, Reijo Sund, Heli Koivumaa-Honkanen, Joonas Sirola, Risto Honkanen, Heikki Kröger, Effectiveness of exercise on fall prevention in community-dwelling older adults: a 2-year randomized controlled study of 914 women, *Age and Ageing*, Volume 52, Issue 4, April 2023, afad059, https://doi.org/10.1093/ageing/afad059

In the originally published version of this manuscript, Table 1 was incorrectly duplicated in place of Tables 2 and 3.

This error has been corrected online.

