# Supplementary material for: Effectiveness of exercise on fall prevention in community-dwelling older adults: a 2-year randomized controlled study of 914 women
Source: Age Ageing. 2023 Apr 23;52(4):afad059. doi: 10.1093/ageing/afad059 (PMC10128158; doi:10.1093/ageing/afad059)
Supplement: aa-22-1748-File002_afad059 [file aa-22-1748-file002_afad059.docx]

**Effectiveness of exercise on fall prevention in community-dwelling older adults: A two-year randomized controlled study of 914 women.**

**APPENDIX 1.**

**Clinical measurement protocol**

A dual X-ray absorptiometry (DXA) scan (GE Lunar iDXA, USA) was performed for the total body and left proximal femur (or right if contained implants). Functional measurements included: (1) single-leg stance (SLS) test (two attempts), performed with eyes open using the better foot, time measured for 30 s (<3 s considered as a fail); (2) squat test (one attempt), i.e. the ability to squat down and touch the floor with the fingertips, measured as not able, able to squat down but assistance needed to get up, and able to squat and get up; (3) isometric leg extension strength (three attempts) in Newtons (N) (HUR Labs, Finland); (4) maximal grip strength (three attempts) in kilograms (kg) (Jamar handheld dynamometer, USA); (5) Timed Up and Go test (one attempt) (TUG); (6) postural body sway protocol with eyes open and eyes closed for 30 seconds, in normal stance and semi-tandem stance (one attempt), using a computerized balance platform (HUR Labs BT3, Finland). The postural sway area (mm^2^) was defined by the trace length defined as 90% confidence of the total sway area. No additional verbal motivation was given to the participants during functional measurements.

**Adverse events**

Adverse events are based on self-reports via a phone call or during a study visit. No AE/SAE information was recorded from the control group. In the exercise group, 14 women reported increased joint pain which was the single most common medical reason for discontinuation. Two women reported pre-syncope symptoms (Dizziness, visual "gray out"), and two women experienced angina pectoris-like chest pain during training. All four discontinued the exercise protocol as a precaution and sought further medical consultation later. All aforementioned cases were considered AE’s. No SAEs were reported during the intervention that could be directly related to training sessions.
